# Too Hot to Handle: A Case of Fever of Unknown Origin

**DOI:** 10.7759/cureus.20942

**Published:** 2022-01-04

**Authors:** Rina R Joshi, Kevin J Hess, Devin M Sullivan, Michael Maguire, Ajeetpal S Hans

**Affiliations:** 1 Internal Medicine, Christiana Care Health System, Newark, USA; 2 Internal Medicine-Pediatrics, Christiana Care Health System, Newark, USA

**Keywords:** double-expressor, mediastinal lymphadenopathy, inguinal lymphadenopathy, diffuse large b cell lymphoma (dlbcl), syndrome of fever of unknown origin

## Abstract

Fever of unknown origin (FUO) is defined as a fever higher than 38.3ºC for at least three weeks. It remains a difficult diagnostic challenge and it carries well over 200 differential diagnoses, including infectious, rheumatologic and malignant etiologies. A methodological approach with clinical deductive reasoning and value-based investigative work-up can establish the diagnosis. This case is about a 76-year-old male with a past medical history of atrial fibrillation, bladder cancer treated with chemotherapy (now in remission) and hydronephrosis with recent ureteropelvic junction stent placement. He presented to the emergency department (ED) for worsening shortness of breath (SOB), weakness, and fevers. His initial workup was notable for a urinary tract infection which was treated with ceftriaxone. However, there was only a limited improvement in the fever. Diagnostic imaging was negative on initial review. He was evaluated by consultants of different specialities including infectious disease, rheumatology, and hematology. Ultimately, the decision was made to discharge the patient home on steroids with further outpatient workup. He returned four weeks later with worsening fever and was found to have new-onset mediastinal lymphadenopathy. A biopsy of an inguinal lymph node was obtained which showed high grade-B cell lymphoma. The patient was continued on prednisone and started on chemotherapeutic agents which included vincristine, rituximab and cyclophosphamide. Shortly after starting treatment, the patient and family elected for hospice. This case demonstrates the importance of continuously questioning the diagnosis at hand and of keeping an open mind when evaluating a patient with FUO.

## Introduction

Fever of unknown origin (FUO) is defined as (1) a temperature greater than 38.3°C (101°F) on several occasions, (2) more than three weeks' duration of illness, and (3) failure to reach a diagnosis despite one week of inpatient investigation [1]. It remains a difficult diagnostic challenge and carries well over 200 differential diagnoses, including infectious, rheumatologic and malignant etiologies [2]. A methodological approach with clinical deductive reasoning and value-based investigative work-up can establish the diagnosis. Here we present a 76-year-old gentleman with complex comorbidities and an intriguing fever of unknown origin.

## Case presentation

A 76-year-old male with a past medical history of high-grade papillary urothelial cell bladder carcinoma treated with Bacillus Calmette-Guerin (BCG) chemotherapy (now in remission), remote history of ischemic stroke with residual left-sided foot drop, atrial fibrillation on anticoagulation with apixaban, heart failure with reduced ejection fraction, and hydronephrosis with recent ureteropelvic junction stent placement who initially presented to the emergency department (ED) for worsening shortness of breath (SOB), weakness, and fevers. His fevers would typically peak at midnight every day and would be associated with altered mental status. History and physical examination did not yield localizing features. Home medications were reviewed, which included carvedilol, lisinopril, famotidine, and hydrochlorothiazide. Initial labs showed lymphopenia with a white blood cell count of 3.2 without bandemia, neutrophil count of 76.9%, and a lactic acid of 4.8 mmol/L. The comprehensive metabolic panel was unremarkable. Urinalysis was positive for leukocyte esterase only. Other notable labs were elevated C-reactive protein (CRP) to 61.5 mg/L, IL-6 of 39.6 pg/mL and ferritin of 1743 ng/mL, with negative COVID-19 and respiratory polymerase chain reaction (PCR). He was initially treated with ceftriaxone for a possible urinary tract infection (UTI). Blood cultures returned negative but urine cultures grew *Enterococcus faecalis*. Antibiotic therapy was switched to vancomycin and later transitioned to oral amoxicillin to finish a 14-day course. Despite adequate treatment of the UTI, the patient continued to spike fevers which prompted a further workup. The primary medical team revisited the whole case, including a thorough review of the patient’s history and physical as well as travel history, previous occupations and family history, all of which were non-contributory. 

**Figure 1:**
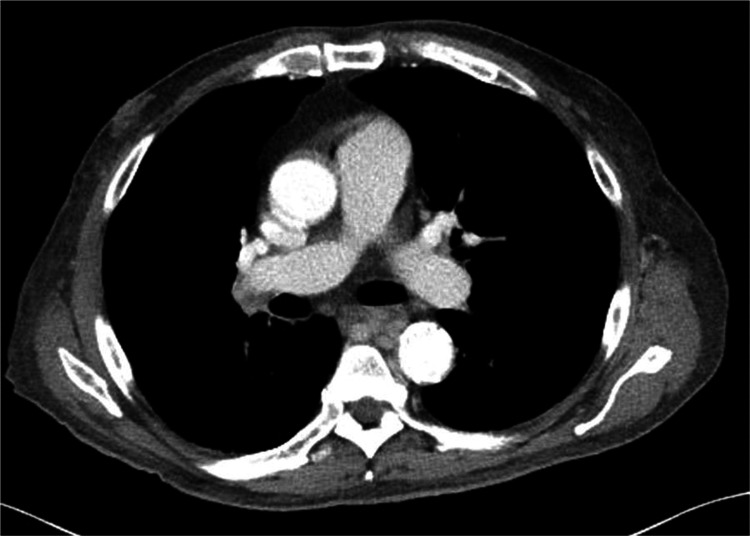
A CT scan of patient's chest on initial admission This computed tomography (CT) imaging was taken during the patient's first admission, which did not show any mediastinal lymphadenopathy.

The patient was hospitalized for approximately one month, during which time additional testing was conducted, including repeat blood cultures, which were negative. Lower extremity dopplers were ordered to evaluate for a deep venous thrombosis and a computed tomography angiography (CTA) of chest/abdomen/pelvis was performed to rule out a pulmonary embolism and malignancy, both of which were also negative (Figure 1). 

As no etiology for the continuing fevers spikes had been identified, rheumatologic evaluation was initiated with anti-neutrophilic cytoplasmic antibodies (ANCA) and rheumatoid factor, which were also negative. Other initial labs included an anti-nuclear antibody, which was less than 1:80, erythrocyte sedimentation rate (ESR) of 140 mm/hr, lactate dehydrogenase (LDH) of 310 unit/L and ferritin of 815 ng/mL. Human immunodeficiency virus (HIV) and chronic hepatitis panel were nonreactive. Infectious disease recommended monitoring the patient off of antibiotics. Hematology was consulted in the absence of a diagnosis to look for a smoldering hematological malignancy. Given the absence of obvious lymphadenopathy or significant cytopenia and normal-appearing peripheral blood smear, a neoplastic etiology was lower on the differential. The patient and family were hoping for diagnosis and possible discharge. Therefore, he was started on empiric corticosteroids as this helped with improvement in his fevers and was subsequently discharged home. The ultimate plan was to taper steroids with outpatient hematology follow up. 

**Figure 2:**
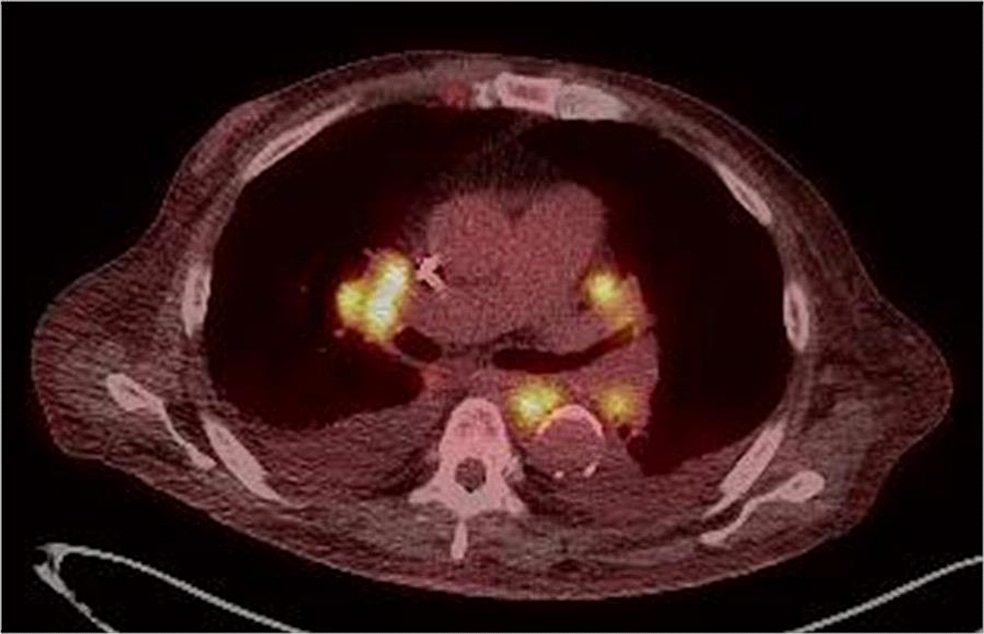
The yellow highlighted regions on this patient's PET/CT represent areas of lymphadenopathy concerning for malignancy PET/CT: positron emissions tomography/computed tomography

However, the patient returned four weeks later with worsening fevers. He had missed all of his outpatient follow-up appointments and had been having fevers again while he was on the final days of his steroid taper. In addition to repeat fevers, the patient was also again reporting shortness of breath. In the ED, a repeat CTA chest was performed, which showed multiple enlarged mediastinal lymph nodes measuring 14 x 22 mm, 1.8 x 1.9 cm and 1.2 x 1.2 cm. Hematology felt that a bone marrow biopsy was warranted given the diagnosis of FUO with a normal LDH. Bone marrow biopsy showed no evidence of monoclonality or aberrant antigen expression and no increase in blasts. A positron emissions tomography/computed tomography (PET/CT) was ordered which showed multiple regions of lymphadenopathy in the inguinal and intra-abdominal region with increased cellular activity, concerning for malignancy (Figure 2). 

An inguinal lymph node biopsy was performed, which showed diffuse large high-grade B-cell lymphoma (DLBCL), non-germinal center type, with double expressor (MYC, BCL-2) status as well as splenic/hepatic lesions. To exclude central nervous system involvement, a magnetic resonance imaging of the brain and magnetic resonance angiography head/neck were performed and were unremarkable. The patient was continued on prednisone and started on chemotherapeutic agents which included vincristine, rituximab and cyclophosphamide. Shortly after starting treatment, the patient began experiencing worsening heart failure, renal failure, and was experiencing rapid functional decline. Ultimately a family meeting was held, where the patient and family elected for hospice.

## Discussion

Fever of unknown origin (FUO) can be difficult to diagnose due to its extensive differential. It is imperative that once a patient is given the diagnosis of FUO, the initial workup should be to rule out infectious processes. Once potential infectious causes have been exhausted, it is feasible to consider autoimmune and malignant etiologies. Our patient underwent an extensive workup for several months, including multiple admissions, until he was finally diagnosed. DLBCL requires precision to diagnose properly given its rapidly progressive nature. In a case report by Onweni et al., the patient had undergone workup for approximately two years. He was initially misdiagnosed with adult-onset Still's disease and had multiple lymph node biopsies that were inconclusive. However, the patient was eventually diagnosed with DLBCL [3]. The etiology for FUO can be difficult to ascertain, possibly leading to incorrect or missed diagnoses. Therefore a systematic and thoughtful approach should occur when working up patients with FUO.

In this case, the patient's initial computed tomography (CT) scan on the first admission did not show lymphadenopathy, and he was discharged home on empiric steroids with improvement in symptoms. However, upon decreasing the dose of the steroids, the patient's fevers reoccurred. Repeat CT a month later did show vast mediastinal lymphadenopathy, although bone marrow biopsy was negative. Given the negative autoimmune and infectious workup, and the patient's prior history of bladder cancer, further workup for malignancy was pursued. The patient's lymphadenopathy was concerning for nodal and extranodal spread. Extranodal versus nodal spread is a large prognostic indicator of mortality. Patients with extranodal spread, elevated LDH, and age greater than 60 are associated with higher mortality [4]. 

Another cause for concern given the patient’s months of fevers is that this patient was diagnosed with papillary urothelial carcinoma in 2016. Although there may not be a direct correlation as the patient did undergo chemotherapy with BCG, there should be strong consideration that the patient's history of urothelial carcinoma with P53 expression may have been a factor in his eventual diagnosis of double-hit lymphoma with expression of MYC and BCL-2 [5]. In a multi-series case review, it was determined that those with B-cell lymphoma with P53 expression had worse overall survival than without it [5]. Additionally, the combination of P53 and MYC expression increased the negative prognostic effect. In this case, there is a possibility that the patient’s prior P53 expression alone increased his chances of developing MYC and BCL-2 markers.

## Conclusions

Fever of unknown origin is a challenging presentation with a large differential. In addition to detailed history taking, a persistent and expansive workup is often required. In this case, the patient’s onset of FUO was sudden and his disease process rapidly progressed. Given this patient’s age and negative initial workup, suspicion for malignancy was high. DLBCL can be a devastating diagnosis despite adequate treatment. This patient may have been predisposed to developing the double-hit mutation given his history of bladder cancer. This case highlights the importance of continuously questioning the diagnosis at hand and keeping an open mind when evaluating a patient with FUO. 
